# Local production of neurostradiol affects gonadotropin‐releasing hormone (GnRH) secretion at mid‐gestation in *Lagostomus maximus* (Rodentia, Caviomorpha)

**DOI:** 10.14814/phy2.13439

**Published:** 2017-10-16

**Authors:** Santiago E. Charif, Pablo I. F. Inserra, Alejandro R. Schmidt, Noelia P. Di Giorgio, Santiago A. Cortasa, Candela R. Gonzalez, Victoria Lux‐Lantos, Julia Halperin, Alfredo Daniel Vitullo, Verónica B. Dorfman

**Affiliations:** ^1^ Centro de Estudios Biomédicos Biotecnológicos Ambientales y Diagnóstico (CEBBAD) Universidad Maimónides Ciudad Autónoma de Buenos Aires Argentina; ^2^ Consejo Nacional de Investigaciones Científicas y Técnicas CONICET Ciudad Autónoma de Buenos Aires Argentina; ^3^ Laboratorio de Neuroendocrinología Instituto de Biología y Medicina Experimental IByME‐CONICET Ciudad Autónoma de Buenos Aires Argentina

**Keywords:** Estradiol, GnRH, *Lagostomus maximus*, LH, reproduction

## Abstract

Females of the South American plains vizcacha, *Lagostomus maximus*, show peculiar reproductive features such as massive polyovulation up to 800 oocytes per estrous cycle and an ovulatory process around mid‐gestation arising from the reactivation of the hypothalamic–hypophyseal–ovary (H.H.O.) axis. Estradiol (E_2_) regulates gonadotropin‐releasing hormone (GnRH) expression. Biosynthesis of estrogens results from the aromatization of androgens by aromatase, which mainly occurs in the gonads, but has also been described in the hypothalamus. The recently described correlation between GnRH and ER *α* expression patterns in the hypothalamus of the vizcacha during pregnancy, with coexpression in the same neurons of the medial preoptic area, suggests that hypothalamic synthesis of E_2_ may affect GnRH neurons and contribute with systemic E_2_ to modulate GnRH delivery during the gestation. To elucidate this hypothesis, hypothalamic expression and the action of aromatase on GnRH release were evaluated in female vizcachas throughout pregnancy. Aromatase and GnRH expression was increased significantly in mid‐pregnant and term‐pregnant vizcachas compared to early‐pregnant and nonpregnant females. In addition, aromatase and GnRH were colocalized in neurons of the medial preoptic area of the hypothalamus throughout gestation. The blockage of the negative feedback of E_2_ induced by the inhibition of aromatase resulted in a significant increment of GnRH‐secreted mass by hypothalamic explants. E_2_ produced in the same neurons as GnRH may drive intracellular E_2_ to higher levels than those obtained from systemic circulation alone. This may trigger for a prompt GnRH availability enabling H.H.O. activity at mid‐gestation with ovulation and formation of accessory corpora lutea with steroidogenic activity that produce the necessary progesterone to maintain gestation to term and guarantee the reproductive success.

## Introduction

The South American plains vizcacha, *Lagostomus maximus*, a hystricognath fossorial rodent that inhabits the Pampean region in Argentina (Jackson et al. 1996), has been reported to show peculiar reproductive features such as preovulatory follicle formation during the 155‐day long‐lasting pregnancy with an ovulatory process around mid‐gestation that produces a considerable number of secondary corpora lutea with oocyte retention, and the consequent increment in circulating progesterone (P4) levels (Jensen et al. 2008; Dorfman et al. 2013). In contrast to most mammals, female vizcacha shows massive polyovulation of up to 800 oocytes per estrous cycle, the highest ovulatory rate so far recorded for a mammal (Weir 1971a,b; Jensen et al. 2006). Massive ovulation results from an unusual constitutive suppression of apoptosis that precludes intraovarian oocyte dismissal through follicular atresia (Jensen et al. 2006, 2008; Leopardo et al. 2011; Inserra et al. 2014).

Gonadotropin‐releasing hormone (GnRH), the central regulator of fertility in mammals, is involved in the modulation of the hypothalamic–hypophyseal–ovary (H.H.O.) axis. We have previously shown that GnRH distribution in the hypothalamic areas of the vizcacha, as medial preoptic area (mPOA), suprachiasmatic area, supraoptic area (SON), arcuate nucleus, and medial eminence, is comparable to a variety of other mammalian species (Dorfman et al. 2011; Inserra et al. 2017). Estradiol (E_2_) regulates GnRH expression through its binding to specific receptors. Two nuclear estrogen receptors isoforms, alpha (ER*α*) and beta (ER*β*), have been described to bind E_2_ with similar affinity (Kuiper et al. 1996). Both receptors can bind to estrogen response elements (ERE) localized in the GnRH gene promoter activating GnRH synthesis (Radovick et al. 1991). Estrogen has a bimodal effect on the hypothalamus, with either an inhibitory or stimulatory action on GnRH delivery. A rapid E_2_ increase at the end of follicular phase triggers a stimulatory effect on GnRH surge resulting in the secretion of gonadotropins, follicle‐stimulating hormone (FSH) and luteinizing hormone (LH) from the pituitary gland, which are essential for follicular maturation and ovulation, respectively (Abraham et al. 1972; Simerly 2002). In contrast, during the luteal phase, E_2_ produces a negative feedback over GnRH secretion (March et al. 1979). In human pregnancy, maternal serum E_2_ level increases considerably above preovulatory levels remaining elevated until the end of gestation, inhibiting menstrual cycle (Simpson and McDonald 1981). The ability of estrogen to induce GnRH surge declines with age both in rodent and human females (Shaw et al. 2011). Lack of estrogen in postmenopausal women contributes to the increase in GnRH mRNA levels (Morrison et al. 2006; Shaw et al., 2010).

Biosynthesis of estrogens is due to aromatization of androgens through aromatase (CYP19A1), an enzyme located in the endoplasmic reticulum and mitochondria (De Montellano 2005). Testosterone (TT) and androstenedione (A4) are converted into E_2_ and estrone (E_1_) by aromatase, respectively. In addition, E_1_ is converted into E_2_ through 17*β*‐hydroxysteroid dehydrogenase type I and A4 is converted into TT by a 17*β*‐hydroxysteroid dehydrogenase type III (Luu‐The et al. 1995; Conley and Hinshelwood 2001). E_2_ is mainly produced by ovaries and it crosses the hematoencephalic barrier; however, the synthesis of E_2_ in the hypothalamus has been also described, suggesting that neurosteroidogenesis is needed for LH surge to induce ovulation and luteinization (Naftolin et al. 1972, Roselli et al. 2009). Brain neuroendocrine areas involved in the control of reproduction have shown aromatase expression and activity (Selmanoff et al. 1977; Roselli and Resko 1987, 1993) suggesting the importance of local synthesis of E_2_, called neuroestradiol, on reproduction.

The peculiar reproductive cycle of the South American plains vizcacha suggests a particular modulation of its neuroendocrine activity to enable ovulation at mid‐gestation. We have recently described a correlation between GnRH and ER*α* expression in the hypothalamus of the vizcacha during pregnancy and the expression of ER*α* in the same neurons that synthetize GnRH, and that these observations occur together at mid‐gestation with preovulatory follicle development and secondary corpora lutea formation, suggesting E_2_ feedback effects on GnRH surge during gestation (Inserra et al. 2017). These observations prompted us to examine the possible contribution of hypothalamic synthetized E_2_ on GnRH expression and delivery, and its involvement on the ovulation during pregnancy observed in the vizcacha.

## Materials and Methods

### Animals

Adult female plains vizcachas [2.5–3.0 Kg body weight; 2–2.5 years old determined by the dry crystalline lens weight, according to Jackson (1986)] were captured from a resident natural population at the Estación de Cría de Animales Silvestres (ECAS), Villa Elisa, Buenos Aires, Argentina. Animals were captured using live traps located at the entrance of burrows. All experimental protocols concerning animals were conducted in accordance with the guidelines published by the National Institutes of Health (NIH, USA) guide for the care and use of laboratory animals (NIH1985), and were reviewed and approved by the Institutional Committee on Use and Care of Experimental Animals (CICUAE) from Universidad Maimónides, Argentina. Handling and euthanasia of animals were performed in accordance with the NIH guidelines for the care and use of laboratory animals (NIH 1985, CCAC 2002, and CCAC 2003). In order to obtain females at different reproductive stages, captures were planned according to the natural reproductive cycle, previously described by Llanos and Crespo (1952), and on our own previous expertise in the field (Jensen et al. 2006, 2008; Dorfman et al. 2011, 2013; Leopardo et al. 2011; Halperin et al. 2013; Inserra et al. 2014, 2017; Charif et al. 2016). Pregnant vizcachas were captured during the breeding season that lasts from April to August. Gestational age was estimated on the basis of capture time and fetal development, including examination of fetal morphology, Theiler Stages of development, visible implantation sites in early‐pregnant animals, and fetal crown–heel length for mid‐pregnant animals (80–130 mm), according to Leopardo et al. (2011). Early‐pregnant females (EP, *N* = 15) were captured in April, mid‐pregnant females (MP, *N* = 15) in July, and term‐pregnant females (TP, *N* = 15) in August; while nonpregnant females (NP, *N* = 15) were captured in mid‐September after the end of the reproductive season. The ovulatory status was assessed by ovary inspection for the presence of ovulatory stigmata at the time of sacrifice and was correlated with the presence of follicles or corpora lutea in hematoxylin‐eosin‐stained ovary sections. Animals were housed under a 12:12 h low‐light cycle to simulate their natural luminal exposure (low light of 12 watts followed by moon light), and 22 ± 2°C room temperature, with ad libitum access to food (alfalfa, potatoes, and apples) and tap water. Animals were housed for 1 week before euthanasia or before beginning with the hormonal treatment for estral cycle synchronization.

### Tissue collection

Animals were anaesthetized by the intramuscular injection of 13.5 mg/kg body weight ketamine chlorhydrate (Holliday Scott S.A., Buenos Aires, Argentina) and 0.6 mg/kg body weight xylazine chlorhydrate (Richmond Laboratories, Veterinary Division, Buenos Aires, Argentina). Animals were sacrificed by trained technical staff by an intracardiac injection of 0.5 mL/kg body weight of Euthanyl™ (sodium pentobarbital, sodium diphenilhidanthoine, Brouwer S.A., Buenos Aires, Argentina) and brains were immediately removed. Isolated brains were either fixed in cold 4% neutral‐buffered paraformaldehyde (PFA) (Sigma Aldrich Inc., St. Louis, Missouri) for immunohistochemical studies or the whole hypothalami were dissected out to a depth of approximately 4 mm with the following borders: the anterior edge of the optic chiasm, the anterior edge of the mammillary bodies, and the two hypothalamic sulci on either lateral side as Charif et al. (2016). Whole hypothalami were incubated in Krebs‐Ringer buffer for pulsatile studies, or anterior hypothalami (AH) fragments surrounded by the anterior, posterior, and lateral borders of the optic chiasm (oc), including the preoptic area (POA), isolated, quickly frozen, and stored at −80°C for Western blot, RIA, or qPCR assays. Ovaries were also removed and fixed in cold 4% PFA for histological inspection of the ovulatory status and follicle development as previously described (Dorfman et al. 2013). Before performing euthanasia, blood samples were taken by cardiac puncture, centrifuged for 15 min at 3000 *g* and the serum fraction was aliquoted and stored at −20°C.

### Sodium dodecylsulphate polyacrylamide gel electrophoresis (SDS)‐PAGE and Western blotting

AH were homogenized (1:3 w/v) in RIPA buffer (0.1 mol/L phosphate buffer saline (PBS) with 1% Igepal, 0.5% sodium deoxycolate, and 0.1% SDS, pH 7.4), containing 0.1 *μ*mol/L aprotionin, 0.1 *μ*mol/L leupeptin, 0.1 *μ*mol/L pepstatin, and 0.1 mmol/L phenylmethylsulfonyl fluoride (PMSF). All procedures were carried out at 4°C. Homogenates were centrifuged for 30 min at 14,000 g and the supernatants collected. Protein concentration was determined by Bradford method (Bradford 1976) using bovine serum albumin (BSA) as a standard. Equal amounts (20 micrograms) of solubilized proteins were mixed (4:1) with sample buffer (1 mol/L Tris‐HCl with 10% w/v SDS, 30% v/v glycerol, 0.1% w/v bromophenol blue, and 0.15% w/v 2‐mercaptoethanol, pH 6.8) and heated for 3 min at 100°C. Samples were separated on an SDS‐polyacrylamide 10% running gel and 4% stacking gel (29:1 acrylamide:bis acrylamide, Bio‐Rad Laboratories, Hercules, California, USA), with 0.25M Tris‐glycine, pH 8.3, as the electrolyte buffer, in an electrophoresis cell (Mini‐PROTEAN II Electrophoresis Cell, Bio‐Rad Laboratories, Hercules, California, USA). For Western blot analysis, proteins were electrotransferred to a 0.2‐mm polyvinylidene difluoride (PVDF) membrane (Immobilon‐P, EMD Millipore Corporation, Billerica, Massachusetts) at 250 mA for 2 h. For protein identification, membranes were blocked 1 h at room temperature with 5% powdered skim milk in PBS‐containing 0.1% Tween 20. Then, they were incubated overnight at 4°C with anti‐P 450 aromatase rabbit polyclonal IgG (1:1000 dilution, ab18995, Abcam, Cambridge, MA), with well‐documented antigen specificity in mice and vizcacha (Looyenga and Hammer 2006; Gonzalez et al. 2012). Data normalization was performed by incubating (overnight, at 4°C) the same membranes with anti‐*β*‐actin mouse monoclonal IgG (1:6000 dilution, AC‐15, Sigma Aldrich Inc., St. Louis, Missouri) in the same membranes. For immunoreactivity development, membranes were incubated with goat antirabbit IgG‐HRP (1:3000 dilution, Bio‐Rad Laboratories, Hercules, California) for aromatase detection or with goat antimouse IgG‐HRP (1:3,000 dilution, Bio‐Rad Laboratories, Hercules, California) for *β*‐actin determination. For chemiluminiscence development, ECL Plus kit (GE Healthcare Ltd., Amersham Place, Buckinghamshire, United Kingdom) was employed. Membranes were scanned with a ImageQuant 350 Capture Imaging System (GE Healthcare Bio‐Sciences AB, Uppsala, Sweden) and dot blots analyzed with Image‐Pro Plus software (Image‐Pro Plus 6, Media Cybernetics Inc., Bethesda, Maryland). The estimation of the band size was performed using a prestained protein ladder (PageRuler, Fermentas UAB, Vilnius, Lithuania) as molecular weight marker expecting to observe a 55 kDa band for aromatase and a 42 kDa band for *β*‐actin. Results were expressed as the ratio between the relative optical density (ROD) of aromatase and the ROD of *β*‐actin. Five animals per group were tested.

### RNA isolation and quantitative polymerase chain reaction (qPCR)

AH were homogenized with TRIzol (Invitrogen, California, USA) according to the manufacturer's instructions to extract total RNA. RNA concentration was quantified by absorption at 260 nm (Genequant, Amersham Biosciences, England) and its integrity confirmed in a 1% agarose gel (Genbiotech, Argentina) in Tris (0.09 mol/L)–boric acid (0.045 mol/L)–EDTA (0.05 mol/L) (TBE) buffer (pH 8.3). RNA integrity was confirmed when the presence of S28 and S18 rRNA subunits was observed. Three *μ*g of total RNA was treated with 1 *μ*L DNaseI (Invitrogen, California, USA) in 1 *μ*L 10X DNase Reaction Buffer (Invitrogen, California, USA) for 30 min at 37°C, and the reaction was stopped with 1 *μ*L 50 mmol/L EDTA (Invitrogen, California, USA) for 10 min at 65°C. The RNA was reverse transcribed into first‐strand cDNA using 1.5 *μ*L random hexamer primers 50 *μ*mol/L (Applied Biosystems, California, USA), 200U reverse transcriptase (RevertAid™ M‐MuLV, Fermentas, Massachusetts), 4 *μ*L First‐Strand Buffer 5x (Fermentas, Massachusetts), 2 *μ*L dNTP mixture 10 mmol/L (Invitrogen, California, USA), and 0.5 *μ*L RNase inhibitor (Ribolock™, Fermentas, Massachusetts), at a 20 *μ*L final volume reaction. The reverse transcriptase was omitted in control reactions, where the absence of PCR‐amplified DNA indicated the isolation of RNA free of genomic DNA. The reaction was carried out at 72°C for 10 min, 42°C for 60 min, and stopped by heating at 70°C for 10 min. cDNA was stored at −20°C until used. Reverse‐transcribed cDNA (1:10 diluted in DEPC‐treated sterile distilled water) was mixed with 6 *μ*L SYBR Green PCR Master Mix (Applied Biosystems, United Kingdom) for qPCR using 0.3 *μ*mol/L forward and reverse oligonucleotide primers. Primer sequences and cycling parameters are shown in Table 1. Primers for GnRH and GAPDH (glyceraldehyde 3‐phosphate dehydrogenase) were successfully employed in vizcacha by Charif et al. (2016) and Gonzalez et al. (2012) respectively. Primers for aromatase were previously published by Galmiche et al. (2006). The primers for GnRH are intron spanning located in exons 2 and 4 of vizcacha, primers for GAPDH are located in exon 5 of mouse and rat, whereas those for aromatase are located in exons 9 and 10 of mouse and rat mRNAs. Oligonucleotides were obtained from Invitrogen (Life Technologies, California, USA). Quantitative measurements were performed using a Stratagene MPX500 cycler (Stratagene, California, USA). Data were collected from the threshold value, taken at the 72°C extension phase, continuously stored during reaction and analyzed by the complementary computer software (MxPro3005P v4.10 Build 389, Schema 85, Stratagene, California, USA). To confirm specificity of the signal, the results were validated based on the quality of dissociation curves generated at the end of the qPCR runs. Relative gene expression was normalized to that of GAPDH as housekeeping gene. For the assessment of quantitative differences in the cDNA target between samples, the mathematical model of Pfaffl was applied (Pfaffl 2001). An expression ratio was determined for each sample by calculating (E_target_)^∆Cq(target^)/(E_GAPDH_) ^∆Cq(GAPDH)^, where E is the efficiency of the primer set and ∆Cq (quantification cycle) is the difference in the threshold cycle with ∆Cq = Cq_(normalization cDNA)_ ‐ Cq_(experimental cDNA)_. The amplification efficiency of each primer set was calculated from the slope of a standard amplification curve of log (ng cDNA) per reaction versus Cq value (E = 10^−(1/slope)^). Efficiencies of 2.0 ± 0.1 were considered optimal. Six animals were tested per group and each sample was analyzed in triplicate along with nontemplate controls to monitor contaminating DNA. Purity of the amplified products was confirmed by 2% agarose gel electrophoresis (Biodynamics, Buenos Aires, Argentina). The presence of the amplified sequence was detected with an UV transilluminator (Labnet DyNA Light TM‐26, USA). Corresponding gel bands were excised and purified with the MinElute Gel Extraction kit (Qiagen, Hilden, Germany). To confirm aromatase, GnRH and GAPDH identities, purified products were sequenced with a 3130xl Genetic Analyzer (Applied Biosystems, California, USA) by the Genomic Unit of the Biotechnology Institute, Instituto Nacional de Tecnología Agropecuaria (INTA), Buenos Aires, Argentina.

**Table 1:** Employed primers and quantitative PCR (qPCR) cycling parameters

Target	Primers	Cycle conditions	Product
Aromatase	F: 5′ TTTACCCTTGAAAACTTTGAGAAGAAC3′	One cycle of 10 min at 95°C followed by 45 cycles of 15 sec at 95°C, 30 sec at 55°C, 30 sec at 60°C, 30 sec at 72°C	122 bp
	R: 5′GTAACCAGGACAACTTTCATCATCAC3′		
GnRH	F: 5′CAGCACTGGTCCTATGGGTTGCG3′	One cycle of 10 min 95°C followed by 40 cycles of 15 sec at 95°C, 30 sec at 60°C, 30 sec at 72°C	189 bp
	R: 5′TTCCTCTTCAATCAGACGTTCC3′		
GAPDH	F: 5′CCAGAACATCATCCCTGCAT3′	One cycle of 10 min at 95°C followed by 40 cycles of 15 sec at 95°C, 30 sec at 60°C, 30 sec at 72°C	67 bp
	R: 5′GTTCAGCTCTGGGATGACCTT3′		

Aromatase primers sequences were previously used by Galmiche et al. (2006). GnRH primers were employed in vizcacha by Charif et al. (2016) and GAPDH primers by Gonzalez et al. (2012). Product = Amplified product length. bp, base pairs.

### Radioimmunoassay (RIA) for hypothalamic GnRH content detection

AH were homogenized in 100 *μ*L 0.1 N HCl, centrifuged for 30 min at 13,000 g and supernatants were recovered. All procedures were carried out at 4°C. GnRH concentration was determined by radioimmunoassay (RIA) as previously described in mouse, *Mus musculus* (Di Giorgio et al. 2013), and validated in the vizcacha (Dorfman et al. 2013; Charif et al. 2016; Inserra et al. 2017). Briefly, six animals were tested per group and each sample was analyzed in duplicate using anti‐GnRH antiserum [rabbit polyclonal HU60 that recognizes GnRH1 with higher affinity than GnRH2 (Mongiat et al. 2006), final dilution 1:50,000] kindly provided by Dr. Urbanski (Division of Neuroscience, Oregon National Primate Research Center). GnRH was iodinated with ^125^I (NEZ 033H Iodine 125, Perkin Elmer, Life and Analytical Science, Waltham, Massachusetts) by the chloramine‐T method (Greenwood et al.^1963^). Intra‐ and interassay coefficient of variation was 7.2% and 11.6%, respectively. The detectability limit was 1 pg/100 μL. Protein content of each sample was determined by Bradford method (Bradford 1976). Results were expressed as the ratio between GnRH value obtained by RIA and the protein content. Six animals per group were tested.

### Immunofluorescence with confocal microscopy

After removal, brains were coronally sectioned in blocks of 5–7 mm thick, fixed in cold 4% paraformaldehyde (PFA) in 0.1 mol/L PBS (pH 7.4) for 72 h, dehydrated through a graded series of ethanol solutions, and embedded in paraffin. For each specimen, the brain region containing the hypothalamus was entirely cut to serial coronal sections (5 *μ*m thick) and mounted onto coated slides. Sections were dewaxed in xylene and rehydrated through a decreasing series of ethanol solutions (100%, 95%, and 70%). One on every 10 sections was separated to perform classical Nissl technique staining to localize hypothalamic nuclei, according to previous description (Dorfman et al. 2011). Adjacent sections were used for immunohistochemical assays. Antigen retrieval was performed by boiling sections in 10 mmol/L sodium citrate buffer (pH 6) for 20 min, followed by 20 min cooling at room temperature. Then, sections were incubated with a blocking solution containing 10% normal serum in PBS (pH 7.4) for 1 h. Aromatase and GnRH immunoreactivity was detected by incubating slides overnight at room temperature with anti‐P 450 aromatase rabbit polyclonal IgG (1:200 dilution, ab18995, Abcam, Cambridge, MA) together with anti‐GnRH mouse monoclonal IgG (1:200 dilution, MAB5456, EMD Millipore Corporation, Billerica, MA). Their specificity was corroborated in adjacent sections by omission of the primary antibody or by preabsorption of the antibodies with synthetic peptides, luteinizing hormone‐releasing hormone synthetic peptide (LHRH, 10 *μ*g, 1:20 dilution, L7134 Sigma Co, St. Louis, MO) for anti‐GnRH mouse monoclonal IgG MAB5456 or aromatase peptide (ab51924, Abcam, Cambridge, MA) for anti‐P 450 aromatase rabbit polyclonal IgG. Antibodies were separately incubated overnight in a rotator at room temperature followed by centrifugation for 20 min at 15,000 g. Colocalization studies were performed by immunofluorescence technique using Alexa‐Fluor 488 coupled horse antimouse IgG for anti‐GnRH IgG detection and Alexa‐Fluor 555 coupled donkey antirabbit IgG for antiaromatase IgG detection, both purchased to Invitrogen Corp. (Invitrogen Corporation, Carlsbad, California, USA) and used at a 1:250 dilution. Finally, slices were mounted with Vectashield (Vector Laboratories, Burlingame, California, USA). To corroborate specificity of detection, sequential line scanning (lambda strobing mode) was used to eliminate any emission crosstalk between the fluorophores. Control sections using each single antibody were also developed and scanned by the three lasers to verify that emission was detected only in the specific single channel. Five animals were tested per group.

### Image analysis

Similar anatomical areas among animals were selected for the histological analysis. Four sections for each animal were analyzed. Colocalization of aromatase and GnRH was studied by immunofluorescence. Microscope images were captured using a Nikon C1 Plus Laser microscope (Nikon Inverted Research Microscope Eclipse Ti, Nikon Corp., Tokyo, Japan) and analyzed with the EZ‐C1 software (EZ‐C1 Software v3.9, Nikon Ltd., London, United Kingdom). Around 20 cells per section were counted at POA and the percentage of cells showing aromatase and GnRH colocalization was quantified. Adobe Photoshop software (Adobe Photoshop CS5, Adobe Systems Inc., Ottawa, Ontario, Canada) was used for digital manipulation of brightness and contrast when preparing the shown images.

### GnRH pulsatility measured by Radioimmunoassay (RIA)

GnRH pulsatility was measured in vitro as previously described (Catalano et al. 2010; Charif et al. 2016). To synchronize the estrous cycle, nonpregnant females captured in early‐March were induced to ovulate. Vizcachas were injected intramuscularly with 250 IU/day of pregnant mare's serum gonadotropin (PMSG, Novormon 5000, Syntex, Argentina) during 3 consecutive days, followed by a single intramuscular injection of 1000 IU of human chorionic gonadotropin (hCG, Ovusyn, Syntex, Argentina) at the 4th day, according to Charif et al. (2016). Animals were sacrificed 9 days after the first injection at an early luteal phase. The success of ovulatory induction was corroborated at sacrifice by ovary inspection. The treatment was successful in 96% of the treated females. After sacrifice, whole hypothalami were rapidly removed, weighed, placed in gelatin precoated eppendorff tubes with 500 *μ*L Krebs‐Ringer buffer (115 mmol/L NaCl, 4.7 mmol/L KCl, 1.2 mmol/L KH_2_PO_4_, 1.2 mmol/L MgSO_4_, 2.56 mmol/L CaCl_2_, and 20 mmol/L NaHCO_3_; pH 7.4) supplemented with 0.1% bovine serum albumin, 25 mmol/L glucose, and 16 mmol/L HEPES buffer, and refrigerated for at least 1 h. Then, hypothalami were preincubated in 500 *μ*L of supplemented Krebs‐Ringer buffer for 30 min at 37°C, followed by incubation in fresh supplemented Krebs‐Ringer buffer (CTL), or fresh supplemented Krebs‐Ringer buffer with 10 *μ*mol/L Letrozole (L6545, Sigma Aldrich Inc., St. Louis, MO), for 6 h at 37°C. Selected concentration of the aromatase inhibitor letrozole was based on previous results (Sonne‐Hansen and Lykkesfeldt 2005; Lu et al. 2012). During incubation, the medium from each tube was collected at 7.5‐min intervals, replaced with fresh medium, and stored at −20°C. A depolarizing concentration of potassium chloride (KCl, 100 mmol/L) was added to the last tube (30 min) to test tissue viability by identifying a marked peak of GnRH release. GnRH content of each collected medium was determined by RIA as described above. GnRH pulsatile parameters were determined using the computer algorithm Cluster8 developed by Veldhuis and Johnson (1986) (Pulse_XP software, http://mljohnson.pharm.virginia.edu/home.html). A 2 × 2 cluster configuration and a t‐statistic of 2 for the upstroke and down stroke, to maintain false‐positive and false‐negative error rates below 10%, were used as suggested by Martínez de la Escalera et al. (1992). Tissues from five different animals were tested per group.

### ELISA of estradiol and estrone delivered to the medium

To verify the inhibition of aromatase activity by letrozole, both estradiol (E_2_) and estrone (E_1_) content was determined in all the aliquots of the collected medium from the hypothalamic explants. Quantification was performed using EIA Estradiol ELISA Kit (EIA2693, DRG Int., Germany) and EIA Estrone ELISA Kit (EIA4174, DRG Int., Germany) according to the manufacturer's instructions. Direct solid‐phase enzyme immunoassays that detect a range 16–2000 pg/mL of E_2_ or 13–1000 pg/mL of E_1_ were developed. Intra‐ and interassays coefficients of variation were below 10.3% for both measurements. The absorbance of the solutions, including the samples and the experimental blank (medium that has not come in contact with the brain explant), was measured at 450 nm (*μ*Quant Microplate Spectophotometer, Bio‐tek Instruments Inc., Winooski, VT) and inversely related to the concentration of E_2_ or E_1_. Calculation of E_2_ and E_1_ content was made by reference to the respective calibration curve and expressed as pg/mL/hypothalamic weight to normalize steroid content to the weight of the corresponding hypothalamic explant.

### Statistical analysis

Values are expressed as mean ± standard deviation (SD). All the experiments were performed by duplicate. Results were evaluated using *t‐*test for comparisons between two groups, or one‐way analysis of variance (ANOVA) followed by Newman–Keuls and Bonferroni's Multiple Comparison tests was employed for comparisons among more than two groups. Statistical analysis was performed using Prism 4.0 (GraphPad Software Inc., San Diego, California, USA). Differences were considered significant when *P *<* *0.05.

## Results

### Hypothalamic aromatase and GnRH display a similar expression pattern throughout gestation in *L*. *maximus*

**Figure 1:**
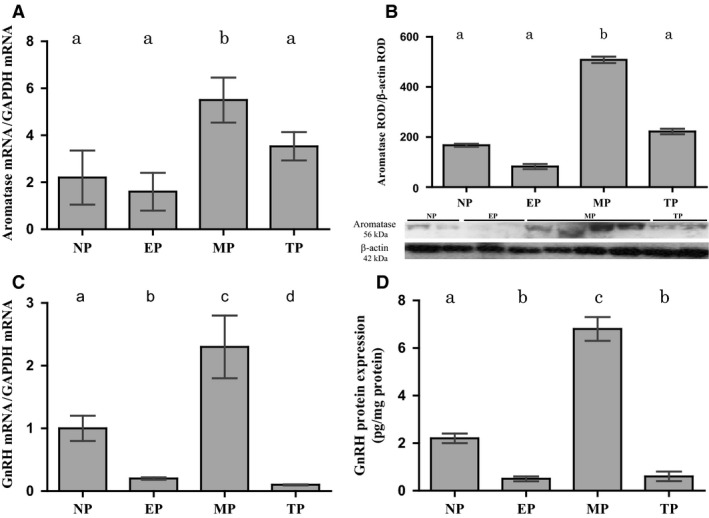
Hypothalamic aromatase expression during gestation is correlated with GnRH expression. Aromatase mRNA and protein expressions normalized to GAPDH and *β*‐actin, respectively, show a significant increase in MP with respect to the other groups (A and B). Representative images of aromatase and *β*‐actin blots obtained by Western blot are shown (B). GnRH mRNA and protein expression, with GAPDH or total protein expression normalization, respectively, show a significant increase in MP when compared to the other groups (C and D). NP: nonpregnant, EP: early‐pregnant, MP: mid‐pregnant, TP: term‐pregnant females, ROD: relative optical density. Different letters in bars indicate significant differences (*P* < 0.05) among groups. *N* = 5 or 6 per group and per technique.

Hypothalamic aromatase expression varied significantly throughout gestation. A significant increase in mid‐pregnant vizcachas compared to nonpregnant, early‐pregnant, and term‐pregnant females was observed, and this profile was consistent at both mRNA and protein levels (Fig. 1A–B). On the other hand, GnRH displayed a pattern similar to that of aromatase; a significant sharp increment at mid‐pregnancy that fell down at term pregnancy reaching values close to those exhibited by early‐pregnant females was observed. Both mRNA and protein values showed a similar pattern of variation throughout gestation (Fig. 1C–D).

The analysis of the aromatase PCR product sequence of *L. maximus* was performed using the Blast algorithm (NCBI, NIH, USA). It showed 96% homology with rat (*Rattus norvegicus*), 93% with mouse (*Mus musculus*), 91% with naked mole‐rat (*Heterocephalus glaber*), and 90% with Chinese hamster (*Cricetulus griseus*) and guinea pig (*Cavia porcellus*).

### Aromatase and GnRH colocalize in the hypothalamus of *L. maximus*

**Figure 2:**
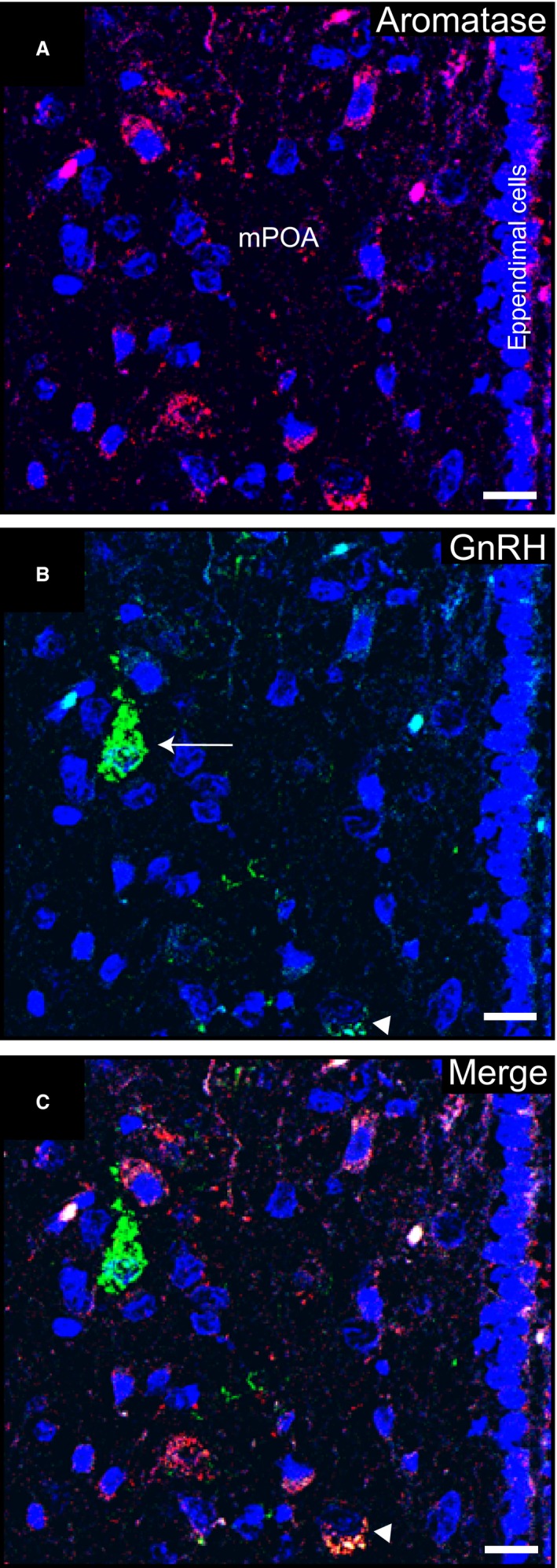
Immunolocalization of aromatase and GnRH in the medial preoptic area of a nonpregnant vizcacha. (A) Representative images of the medial preoptic area (mPOA) showing cytoplasmic aromatase immunoreactive neurons (red). (B) GnRH immunoreactive neurons (green) in the same optic area shown in A with low (arrowhead) or high (arrow) GnRH labeling. (C) Merged image showing expression of aromatase in a GnRH low‐expressing neuron (arrowhead), and a GnRH high‐expressing neuron (green) surrounded by aromatase‐expressing neurons (red). Nuclei were counterstained with DAPI (blue). Scale bar: 10 *μ*m. *N* = 5.

Cytosolic aromatase and GnRH localization was determined in neurons of the mPOA of nonpregnant females (Fig. 2A–B). Two subgroups of GnRH neurons, high‐ or low‐labeled, were found (Fig. 2B). Qualitatively, low‐labeled neurons were more abundant than high‐labeled neurons, being the latter scattered throughout the mPOA. Strikingly, neurons with high signal for GnRH did not show aromatase expression while neurons with low density of GnRH depicted cytoplasmic aromatase colocalization (Fig. 2C). In addition, randomly distributed GnRH immunopositive axonal varicosities were observed (not shown). Aromatase‐ or GnRH‐specific labeling was not detected after preabsorption of the primary antibodies with aromatase or LHRH synthetic peptides, in adjacent sections, or after omission of the primary antibodies (not shown).

**Figure 3:**
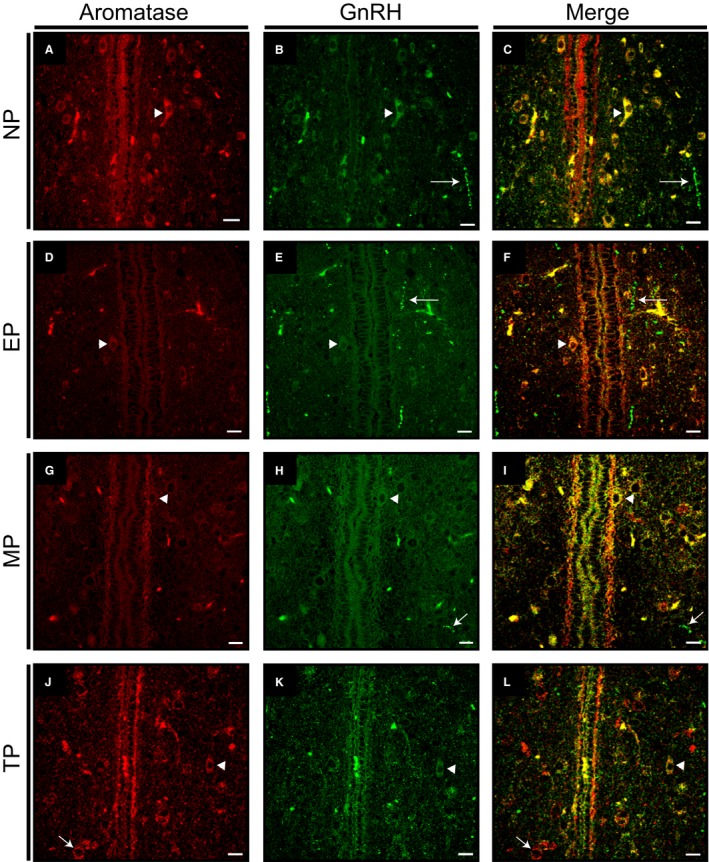
Aromatase and GnRH colocalization in the medial preoptic area of the hypothalamus throughout different reproductive stages. (A, D, G, J) Representative images of the medial preoptic area (mPOA) showing cytoplasmic aromatase immunoreactive neurons (red, arrowhead). (B, E, H, K) Low labeling GnRH immunoreactive neurons (green, arrowhead) in the same optic area shown in A, D, G, and J, respectively. (C, F, I, L) Merged images showing expression of aromatase in GnRH low‐expressing neurons (yellow, arrowhead) and in neurons lacking GnRH immunoreactivity (red). (L) Neurons showing only aromatase expression could be observed at term pregnancy (red, arrow). GnRH immunoreactive axonal varicosities distributed in the mPOA could be observed in all groups (arrows). NP, nonpregnant; EP, early‐pregnant; MD, mid‐pregnant; TP, term‐pregnant vizcachas. *N* = 5 per group. Scale bars: 10 *μ*m

**Figure 4:**
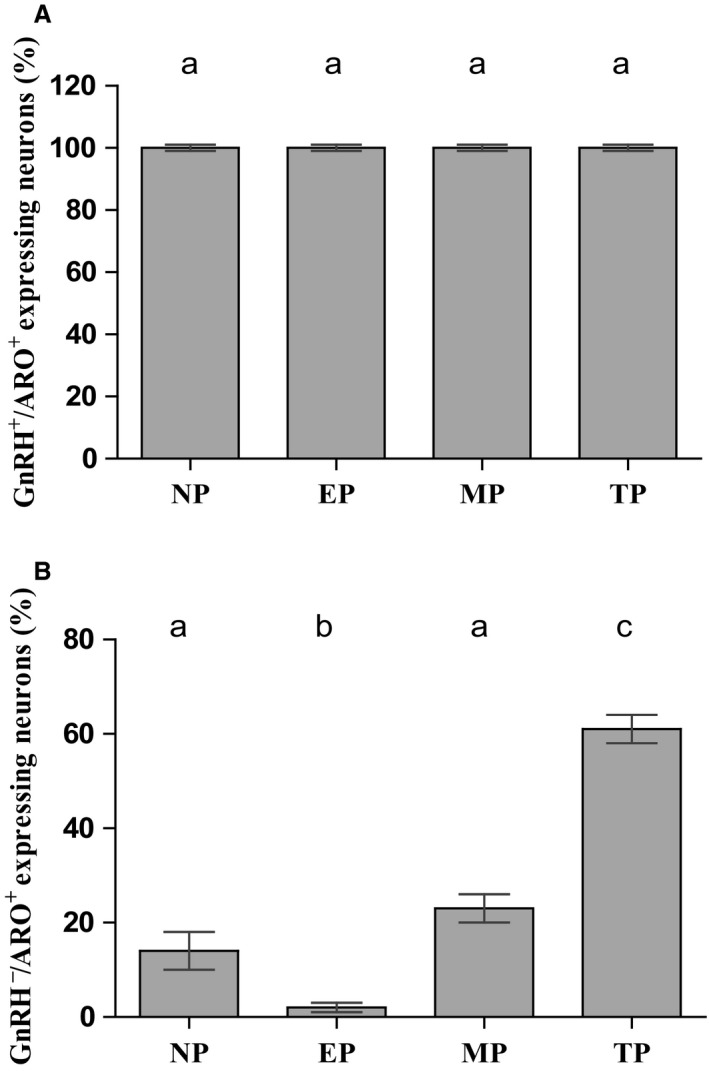
Quantification of cells with aromatase and GnRH colocalization. (A) Quantification of aromatase and GnRH colocalization in cells of POA showing no differences in the percentage of GnRH cells with aromatase immunoreactivity throughout the reproductive cycle (GnRH+/ARO+). (B) Significant increase in the percentage of aromatase cells without GnRH immunoreactivity with pregnancy progression (GnRH ^−^/ARO+). Different letters indicate significant differences among groups with *P* < 0.05. NP, nonpregnant; EP, early‐pregnant; MP, mid‐pregnant; TP, term‐pregnant females. *N* = 5 per group.

The colocalization of aromatase and GnRH in the mPOA was detected in all experimental groups (Fig. 3). All the low‐density GnRH immunoreactive neurons showed aromatase expression without significant variations among the evaluated groups (Fig. 4A). However, additional aromatase immunoreactive neurons lacking GnRH immunolabeling were also observed (Fig. 3I, L). The abundance of aromatase‐expressing cells lacking GnRH expression showed significant differences throughout gestation, increasing from less than 5% at early gestation to approximately 60% at term pregnancy (Fig. 4B).

### Neuroestradiol is involved in GnRH hypothalamic release

**Figure 5:**
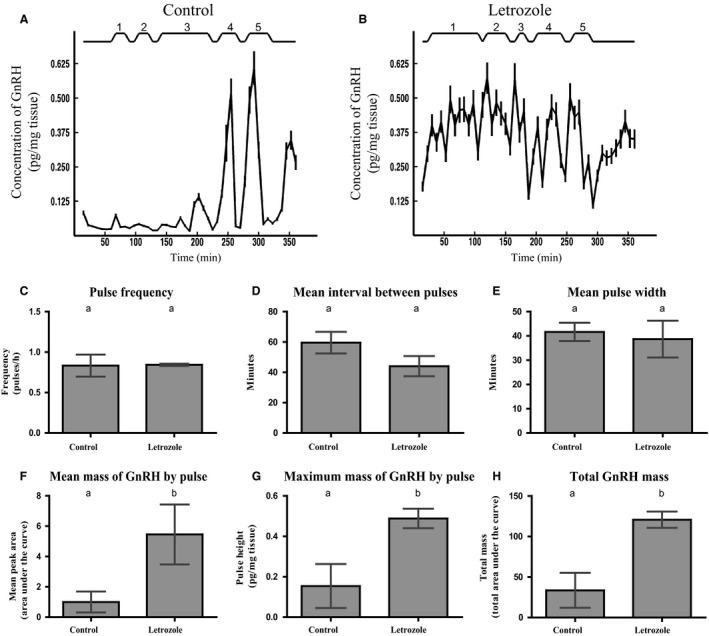
Neuroestradiol is involved in GnRH hypothalamic release. Representative GnRH pulsatile profiles from female hypothalamic explants control (A) and supplemented with letrozole (B), determined by radioimmunoassay (RIA). Five pulses were detected in both conditions during 6 h of treatment. The number of each pulse is indicated above the graph, whereas the bottom line indicates the time lapse of the GnRH release study. Several pulsatil parameters were evaluated: Pulse frequency (C), mean interval between pulses (D), mean pulse width (E), mean mass of GnRH delivered by pulse (mean area of the peaks (F), the maximum mass of GnRH secreted by pulse (mean pulse height), (G), and the total GnRH mass secreted during the 6 h of the experiment (under curve area), (H). Significant increase was determined in maximum mass of GnRH delivered by pulse**,** the mass of GnRH secreted by pulse**,** and total GnRH delivered in letrozole‐supplemented hypothalamus related to control. Different letters indicate significant differences between groups with *P* < 0.05. *N* = 5 per group.

**Figure 6:**
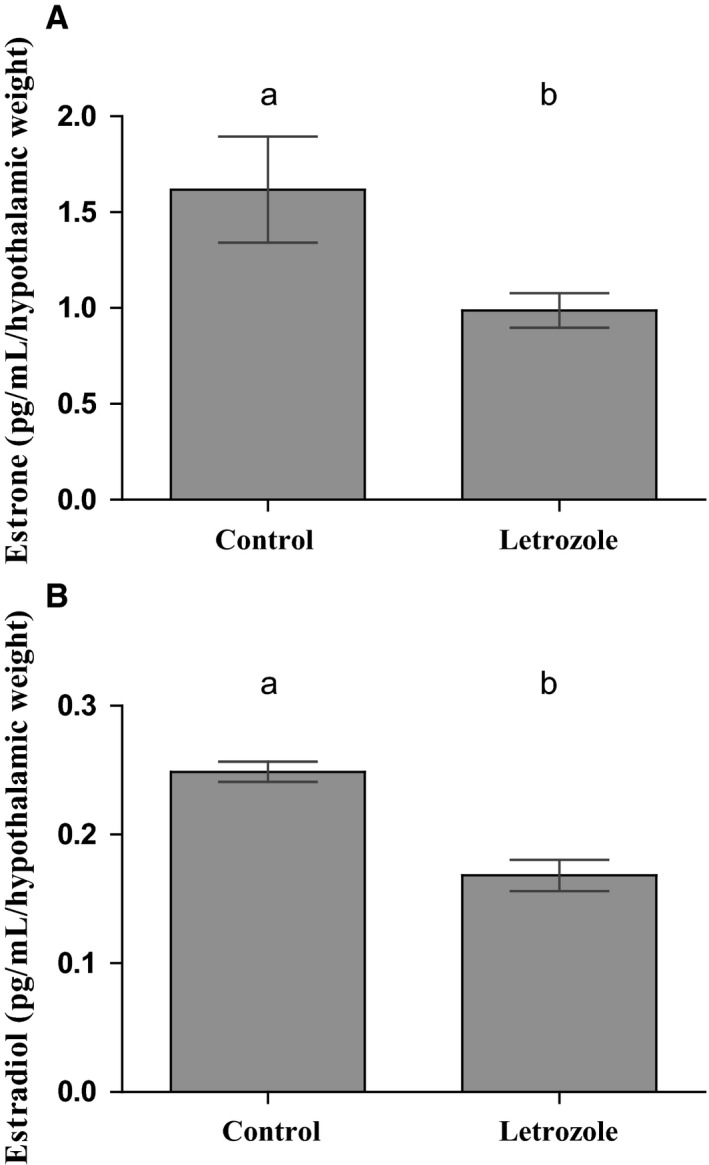
Hypothalamic synthesis and delivery of estrone and estradiol is decreased by letrozole. Hypothalamic synthesis and delivery of E_1_ (A) and E_2_ (B) was significantly decreased by inhibition of aromatase activity as a result of treatment with the specific inhibitor letrozole. E_1_ and E_2_ content was expressed as pg/mL/hypothalamic weight in order to normalize steroid content to the weight of the corresponding hypothalamic explant. For each evaluated group, values represent the average of E_1_ or E_2_ in the incubation media corresponding to the aliquots where each GnRH pulse showed its maximum height. Different letters indicate significant differences between groups with *P* < 0.05. *N* = 5 per group.

GnRH delivery by hypothalamic explants supplemented with the aromatase inhibitor letrozole was evaluated. GnRH pulsatile pattern in letrozole‐treated explants was compared against its basal pulsatility in hypothalamic explants without letrozole (Control) throughout six hours long. Both letrozole treatment and control condition showed five pulses of GnRH throughout the experiment (Fig. 5A–B). Parameters concerning the GnRH pulsatile delivery (Fig. 5C–E) and the GnRH‐secreted mass (Fig. 5F–H) were evaluated. Pulsatile parameters concerning GnRH delivery as pulse frequency, mean intervals between pulses, and mean pulse width did not show significant variations between letrozole and control conditions (Fig. 5C–E respectively). In contrast, a significant increase was observed in the GnRH‐secreted mass when hypothalamic explants were supplemented with letrozole versus control condition. This parameter is reflected in the mean mass of GnRH delivered by pulse (Fig. 5F), the maximum mass of GnRH secreted by pulse (Fig. 5G), and the total GnRH mass secreted during the six hours of the experiment (Fig. 5H). To confirm that letrozole inhibited the aromatase activity in hypothalamic explants, E_1_ and E_2_ content was determined in all the aliquots corresponding to GnRH peaks collected throughout experiment (Fig. 6). Letrozole induced a significant decrease in E_1_ and E_2_ of hypothalamic origin with respect to control condition (Fig. 6A–B). This confirmed the decrease in aromatase activity induced by letrozole action.

## Discussion

This study shows the first evidence of expression of aromatase enzyme in the mPOA and its hypothalamic activity in *Lagostomus maximus*. Variations in aromatase expression during pregnancy, with a concordant pattern with GnRH expression, suggest the action of neuroestradiol in GnRH expression at mid‐gestation. In addition, two subgroups of GnRH neurons were detected according to their labeling of GnRH by immunohistochemistry. The action of neuroestradiol on GnRH modulation and its possible role in ovulation in the South American plains vizcacha during gestation is described.

Fertility depends on a complex steroid feedback mechanism that regulates the activity of GnRH neurons. E_2_ is known as a major regulator of GnRH neuron activity by bimodal feedback mechanisms. Aromatase is a monooxygenase that belongs to the cytochrome P450 superfamily and is responsible for the aromatization of androgens into estrogens. This enzyme is highly conserved among vertebrates. Its localization and activity in the hypothalamus of several species like rat, monkey, Japanese quail, and zebra fish has been reported (Roselli et al. 1985; Roselli and Resko 1987; Schumacher and Balthazart 1987; Vockel et al. 1990; Naftolin et al. 2001). In this work, we described the localization of aromatase in the mPOA of the hypothalamus of the South American plains vizcacha and its colocalization with GnRH suggesting the direct modulation of neuroestradiol on GnRH expression. Two subpopulations of GnRH neurons have been described in the rostral hypothalamus of rats. They are composed of abundant low‐intensity–stained GnRH neurons (more than 80% of total GnRH neurons) and some scattered highly‐intensity–stained GnRH neurons (Wray et al. 1989). In this work, we show the existence of these both groups in the mPOA of the vizcacha. Rats and mice also show aromatase‐expressing neurons in the neighborhood of GnRH cells in the mPOA (Wagner and Morrell 1996), but not the coexpression of aromatase and GnRH in the same neurons. The cytoplasmic colocalization of GnRH with aromatase in the mPOA of the vizcacha suggests the capacity of these cells to convert testosterone into E_2_, which may directly affect GnRH expression. Strikingly, we noted that this colocalization was exclusively detected in the low‐stained GnRH neurons, whereas highly stained GnRH neurons were devoid of aromatase expression. It has been hypothesized that GnRH neuron population was divided into pulse and surge cells and differences in neuronal activity may depend on E_2_ modulation (Caraty et al. 1998; Boukhliq et al. 1999). In this context, the two subpopulations of GnRH neurons found in the mPOA of the vizcacha may be related with differences in the modulation of pulse and surge of GnRH by E_2_ action. In addition, it seems important to note that both neurons and glia have been described to synthesize E_2_ in vitro (Zwain and Yen 1999), however, in this study aromatase was only localized in neurons. This is in accordance with immunohistochemical studies performed in Japanese quail, rat, monkey, and humans that show neurons as the primarily aromatase‐expressing cells in mammals (Naftolin et al. 1996) while glial expression of aromatase is induced by brain damage with neuroprotective effects (Saldanha et al. 2009).

The action of neuroestradiol‐modifying GnRH release has been previously shown using microdialysis method in monkeys (Kenealy et al. 2013). The changes in GnRH pulsatile parameters induced by aromatase inhibition here described confirm the influence of neuroestrogens on GnRH release. The increased level of pulsatile parameters resulting from blocking aromatase activity with letrozole, confirmed by E_1_ and E_2_ decrease, suggests that GnRH delivery may be negatively affected by a feedback action of these steroids. Concordantly, the positive feedback of estradiol on GnRH could be also confirmed during pregnancy when increased levels of E_2_ at mid‐gestation are correlated with increased levels of hypothalamic GnRH content and serum LH (Inserra et al. 2017).

The bimodal effect of E_2_ over GnRH during the estrous cycle of mammals appears to occur at different anatomical localization and it is generally accepted that it is mediated by kisspeptin in mice. This peptide mediates the E_2_‐negative feedback in the arcuate nucleus and its positive feedback in the anteroventral periventricular nucleus (Smith et al. 2006). Previous unpublished observations of our group on kisspeptin indicate that GnRH^+^/RE^+^ hypothalamic cells of nonpregnant vizcachas show also GPR54 kisspeptin receptor reinforcing kisspeptin involvement on E_2_ modulation (Schmidt A. R. et al. unpubl. results). However, GPR54 knockout mice retain the ability to generate LH surge (Dungan et al. 2007) suggesting alternative ways of GnRH delivery modulation. Other factors such as the neuromodulators *γ*‐aminobutiric acid (GABA) and glutamate may be also involved (Romanò et al. 2008). In line with this, the negative feedback exerted by E_2_ over its own receptor decreases GnRH dynamics. ER expression changes during the estrous cycle, being decreased during the proestrous stage, when E_2_ levels are the highest (Morrison et al. 2006). In addition, several in vivo and in vitro studies developed in rat and mouse have shown that estrogen decreases GnRH gene expression (Zoeller and Young 1988; Wray et al. 1989; Wolfe et al. 1996; Spratt and Herbison 1997). The action of E_2_ on GnRH neurons through ER*α* has been checked over for approximately 40 years by a variety of histological methods. Most data indicate that in a large variety of mammalian species like mink, sheep, ewe, guinea pig, mouse, and rat, GnRH neurons do not express ER*α* but does ER*β* (Watson et al. 1992; Herbison et al. 1993; Laflamme et al. 1998; Warembourg et al. 1998; Herbison and Pape 2001; Smith and Jennes 2001; Wintermantel et al. 2006). However, some works showing ER*α* expression in GnRH neurons of the POA of rats (Butler et al. 1999) and in GT1‐7 and GN11 cell lines that express GnRH have been reported (Shen et al. 1998; Roy et al. 1999; Ng et al. 2009). Adding to the controversy, we have recently shown ER*α* expression in GnRH neurons of medial POA and SON of the South American plains vizcacha (Inserra et al. 2017). It was hypothesized that the direct action of E_2_, by means of ER*α* on GnRH neurons, may represent a differential reproductive strategy of vizcacha to guarantee GnRH synthesis during pregnancy where GnRH neurons may sense circulating estradiol (Inserra et al. 2017). Concordantly, the locally produced E_2_ by aromatase, in the same neurons where ER*α* is expressed, may contribute to the direct regulation on GnRH synthesis and delivery allowing ovulation during gestation.

Both genomic and nongenomic actions of estradiol interact to induce responses. Estradiol may act by classical and nonclassical receptors. Classical receptors as ER*α* and ER*β* were described to be located in the cell nucleus with transcriptional effects, or to be embedded in cell membranes with rapid effects as the control of intracellular calcium oscillations in GnRH neurons (Romanò et al. 2008). In mouse, for example, it was described that E_2_‐induced increase in intracellular calcium concentration stimulates firing activity of GnRH neurons through ER*β* (Sun et al. 2010). In addition, nonclassical receptors as GPR30 and ER‐X localize in the plasma membrane and can rapidly activate a wide variety of intracellular signaling pathways like modulations of intracellular calcium concentration and rapid protein phosphorylation in several tissues including hypothalamus (Gu et al. 1996; Mermelstein et al. 1996; Moss et al. 1997; Ábrahám et al. 2004). High doses of estradiol are required to activate nongenomic responses in relation with doses required to induce genomic effects (Cornil et al. 2006). Considering that aromatase activity is markedly affected by calcium‐dependent phosphorylation (Balthazart et al., 2003) and that it may be induced by E_2_ nongenomic effects, actions of ER*α* in the vizcacha may be involved in changes in aromatase expression during pregnancy directly affecting autoregulation of E_2_ synthesis.

A succession of effects predominates in the E_2_ feedback process with early rapid E_2_ effects followed by classical later genomic responses (Herbison 1998). The increase in brain aromatase activity, which may contribute with a rapid increment of in situ synthetized E_2_, could potentially activate responses that are insensitive to the lower concentrations of estrogens obtained from the peripheral circulation and be the initial step to trigger GnRH synthesis and surge. This idea has been well established in the case of negative feedback (Herbison 2009); however, its critical role on positive feedback actions had not been completely elucidated. Present data contribute with the analysis of both E_2_ regulatory pathways and allow to hypothesize that the synthesis of neuroestradiol might act as the trigger for a prompt GnRH availability enabling H.H.O. activity at mid‐gestation. The activation of H.H.O allows preovulatory follicle formation followed by the development of accessory corpora lutea with steroidogenic activity that produces the necessary progesterone to maintain gestation to term and guarantee the reproductive success.

**In conclusion,** local synthesis of E_2_ by aromatase may drive to higher E_2_ levels than those obtained from systemic circulation alone, with fluctuations in a rapid manner. This could play a crucial role in regulating GnRH delivery being part of the peculiar reproductive strategy of the vizcacha to ensure GnRH availability. The contribution of aromatase activity to GnRH release may represent a fast mechanism for the H.H.O. axis activity regulation.

## Conflict of Interest

None declared.
